# Lack of Difference in Tocilizumab Efficacy in the Treatment of Severe COVID-19 Caused by Different SARS-CoV-2 Variants

**DOI:** 10.3390/jpm12071103

**Published:** 2022-07-04

**Authors:** Oleksandr Oliynyk, Wojciech Barg, Yanina Oliynyk, Serhij Dubrov, Vitaliy Gurianov, Marta Rorat

**Affiliations:** 1 Department of Anaesthesiology and Intensive Care, Bogomolets National Medical University, 01601 Kyiv, Ukraine; alexanderoliynyk8@gmail.com (O.O.); president@aaukr.org (S.D.); 2 Department of Emergency Medicine, Medical College of Rzeszow University, 35-959 Rzeszów, Poland; 3 Department of Human Physiology, Medical College of Rzeszow University, 35-959 Rzeszów, Poland; wbarg@ur.edu.pl; 4 Department of Immunology and Allergology, Bogomolets National Medical University, 01601 Kyiv, Ukraine; kafedra.imun@gmail.com; 5 Department of Medical Statistics, Bogomolets National Medical University, 01601 Kyiv, Ukraine; managementnmu@gmail.com; 6 Department of Forensic Medicine, Wroclaw Medical University, 50-367 Wroclaw, Poland

**Keywords:** mutation, inteleukin-6 inhibitor, ARDS

## Abstract

Tocilizumab (TOC) is presumed to be an effective and safe treatment for severe COVID-19, but its usefulness has not been yet investigated for different SARS-CoV-2 variants. This study aimed to evaluate the influence of TOC on mortality in patients with severe COVID-19 caused by Delta and non-Delta SARS-CoV-2 variants. In a retrospective analysis, we compared the medical records of 78 and 224 patients with severe COVID-19 due to Delta and non-Delta variants, respectively. A total of 30 patients with Delta and 84 with non-Delta variants were treated with TOC in addition to standard therapy. There were no statistically significant differences in mortality rate when comparing Delta vs. non-Delta patients nor when comparing those treated with TOC vs. not treated with TOC in both variants. Using a logistic regression model, in the examined population as a whole, we found an increased (*p* < 0.05) risk of death as leukocyte and erythrocyte counts decreased and as procalcitonin increased. Increased procalcitonin was significant for mortality in the Delta group, while decreased IL-6, leukocytes, and platelets and increased fibrinogen and procalcitonin were significant in the non-Delta group. Tocilizumab efficacy in severe COVID-19 does not differ between Delta or non-Delta virus variants. The Delta variant of SARS-CoV-2 does not increase mortality when compared to other virus strains.

## 1. Introduction

The coronavirus disease 2019 (COVID-19) pandemic caused by the severe acute respiratory syndrome coronavirus 2 (SARS-CoV-2) infection, resulting in over 350 million cases and over 5.5 million deaths by the end of 2021, is a challenge for healthcare systems all over the world [1]. The SARS-CoV-2 infection is biphasic, with an initial phase of virus replication and only mild clinical symptoms, and an inflammatory phase usually with interstitial pneumonia with or without hypoxemia [2]. The majority of patients remain oligosymptomatic or even asymptomatic. However, some of those with severe symptoms develop a life-threatening course of COVID-19 associated with systemic hyperinflammation. The biomarkers of this hyperinflammation are clearly elevated concentrations of inflammatory markers such as D-dimer, ferritin, and C-reactive protein (CRP) and a cytokine storm—an excessive release of pro-inflammatory cytokines, including interleukin-1, interleukin-6 (IL-6), and tumour necrosis factor α [3,4]. It was demonstrated that within the cytokine storm, severe COVID-19 progresses with a significant rise in IL-6 levels [5]. Consequently, anti-Il-6 medications were proposed for the treatment of severe COVID-19, and tocilizumab (TOC) is the one most often used.

Tocilizumab, an IL-6 monoclonal antibody, initially used in the treatment of autoimmune inflammatory diseases, such as rheumatoid arthritis, has been approved in the USA for cytokine release syndrome since 2017 [6]. It has started to be used intensively in the treatment of severe COVID-19. Initial reports on the effectiveness of this therapy in COVID-19 varied significantly, ranging from an evident reduction in the need for mechanical ventilation and death rate, through lack of effect on the course of the disease, to evidence of therapy-related harm. Although, according to most data currently available, TOC is presumed to be beneficial in severe COVID-19, there are no clear indications for its use and no clearly defined biomarkers for the indication of treatment with TOC or precisely identified patients that are most likely to benefit from it [7]. In the very recently updated Infectious Diseases Society of America Guidelines, it is suggested that TOC is added to the standard of care (i.e., steroids), but this recommendation is labelled as conditional, with low certainty of evidence [8].

There is also no data regarding TOC safety and efficacy with respect to SARS-CoV-2 variants. This might be an emerging issue as mutations in the virus could potentially trigger a different inflammatory response. This in turn may alter sensitivity to the treatment used. In the studies conducted so far, the effectiveness of treatment with TOC has not been assessed with regard to the virus variant, which may be of significant clinical importance.

The objective of this research was to assess TOC safety and efficacy in patients with severe COVID-19 and hyperinflammatory syndrome resulting from infection with different SARS-CoV-2 variants.

## 2. Materials and Methods

In a retrospective study, the medical records of 983 consecutive patients with severe COVID-19 hospitalised from 1 August 2020 to 30 October 2021 in the Department of Anaesthesiology and Intensive Care at Kyiv City Clinical Hospital № 4 were examined. Of them, 302 were selected for further analysis.

Inclusion criteria comprised:

1. laboratory-confirmed SARS-CoV-2 infection (positive reverse transcription-polymerase chain reaction, RT-PCR),
2. presence of interstitial pneumonia on a computed tomography (CT) scan,
3. respiratory failure with oxygenation index (PaO2/FiO2 ratio) <300 mm Hg,
4. hyperinflammatory syndrome—the presence of at least two out of three biomarkers for hyperinflammation (increased C-reactive protein >100 mg/L, ferritin >900 µg/L, D-dimer >1500 µg/L) [9].

Exclusion criteria comprised:

1. symptoms of bacterial sepsis defined as leukocyte count (WBC) >10.0 × 10^9^/L and/or procalcitonin (PCT) >2.0 ng/mL and/or positive blood culture,
2. cardiogenic pulmonary oedema,
3. acute brain stroke,
4. advance chronic pulmonary disease,
5. malignancies or any decompensated chronic diseases,
6. pregnancy,
7. participation in other interventional studies.

Patients were assigned to two groups: those with Delta SARS-CoV-2 variant infections (*n* = 78) and those with other SARS-CoV-2 variants (*n* = 224). Both groups were then divided into those who received tocilizumab (30 and 84 patients, respectively) and those who did not (48 and 140, respectively).

Since 1 June 2021, all hospitalised patients were tested for the mutation specific to the SARS-CoV-2 Delta variant. The mutation, characterised as L452R/E484Q/P68IR/E484K, was detected using a Nuclear Acid Extraction-Purification Kit from Sansure Biotech (Sansure Biotech Inc., No 680, Lusong Road, Zuelu District, 410205 Changsha, Hunan Province, China). The determination of the mutation was carried out according to the relevant instructions: Doc.#: S10016E Manual, Doc. Version: V00. Revision Date: 26 August 2020. Patients hospitalized before 1 June 2021 were assigned to the non-Delta group. This criterion was selected based on the analysis of the reports published by the Public Health Center of the Ministry of Health of Ukraine, which conducts epidemiological surveillance in Ukraine by regular molecular testing samples taken from patients from various regions of the country.

Treatment with tocilizumab was started between day 8 and day 14 from the onset of symptoms. Tocilizumab was administered intravenously at a dose of 400 mg for two consecutive days. All patients were treated with methylprednisolone 32 mg or dexamethasone 6 mg daily (ultimately for 10–14 days from admission), low molecular weight heparin administered subcutaneously in prophylactic doses, antibiotics (in case of suspected or confirmed bacterial infection), and balanced fluid therapy. Mortality was assessed over a 28-day period from admission to the intensive care unit (ICU).

### Statistical Analysis

MedCalc^®^ Statistical Software version 2014 (MedCalc Software Ltd., Ostend, Belgium; https://www.medcalc.org; accessed on 20 December 2021) was used for the analysis. If the distribution of values differed from normal by the Shapiro–Wilk test, the median (Me) value and interquartile range (QI-QIII) were calculated to characterise the quantitative measures. The Mann–Whitney test was used for comparison between the two groups. Frequency (%) was calculated for qualitative measures and Fisher’s exact test was used to compare the two groups. The logistic regression analysis with a stepwise method was used to quantify the impact of examined variables on mortality risk. The prognostic quality of the models was assessed using a receiver operating characteristic curve (ROC-curve), the area under the ROC-curve (AUC), and its 95% CI were calculated. The impact of the examined variables was assessed by the odds ratio (OR) values, for which a 95% CI was calculated. A two-tailed test was used in the analysis. For all statistical tests, the *p* value < 0.05 was considered significant.

## 3. Results

Demographic and medical data for the groups studied is shown in Table 1.

There were statistically significant differences between the groups for age, erythrocyte and lymphocyte counts, and respiratory index.

Sixteen factors were presumed to potentially influence the risk of death in coronavirus variants: treatment with TOC, age, sex, body temperature, platelet, leucocyte, lymphocyte, and red blood cell counts, interleukin-6, procalcitonin, ferritin, fibrinogen, C-reactive protein, D-dimer concentration, and respiratory index PaO_2_/FiO_2_. In a multifactor logistic regression model using a stepwise method (entering variable if *p* < 0.05, removing variable if *p* > 0.1), three variables were found to influence the risk of death: erythrocytes, leukocytes, and procalcitonin. There was an increased (*p* < 0.05) risk of death when leukocyte and erythrocyte counts decreased and when procalcitonin levels increased. It is worth noting that neither treatment with TOC nor the virus variant influenced the risk of death. Table 2 presents the results of the multifactor logistic regression analysis.

**Figure 1:**
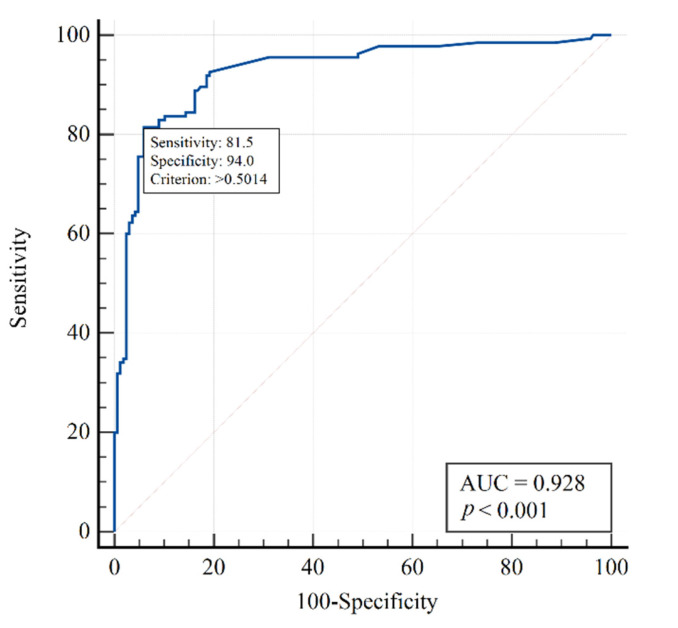
ROC curve for the three-factor model for predicting mortality risk in the entire study population comprising both Delta and no-Delta patients. Multivariate logistic regression model using procalcitonin, erythrocytes, and leukocytes.

As demonstrated in Figure 1 using the ROC curve, the model based on the selected variables is adequate with AUC = 0.928 (95% CI 0.89–0.95). This indicates a very strong relationship between the risk of death and the selected variables.

To estimate the influence of treatment with TOC on mortality in the Delta and non-Delta groups, we first compared basic demographic and medical data with respect to TOC treatment in those groups (Table 3, respectively).

As shown in Table 4, there were no statistically significant differences in the number of deaths in the Delta group and the non-Delta group with respect to treatment with TOC.

To determine the set of factors associated with the risk of death separately for the Delta and the non-Delta groups, again multifactor logistic regression analyses were performed using the same set of factors potentially influencing the risk of death as the one used in the previous analysis. Using the stepwise method (entering variable if *p* < 0.05, removing variable if *p* > 0.1), only increased procalcitonin was found to be significant for the Delta group, while five variables—decreased IL-6, leukocytes, and platelets, as well as increased fibrinogen and procalcitonin—were found to be significant for the non-Delta group. Table 5 and Table 6 demonstrate the results of those analyses.

**Figure 2:**
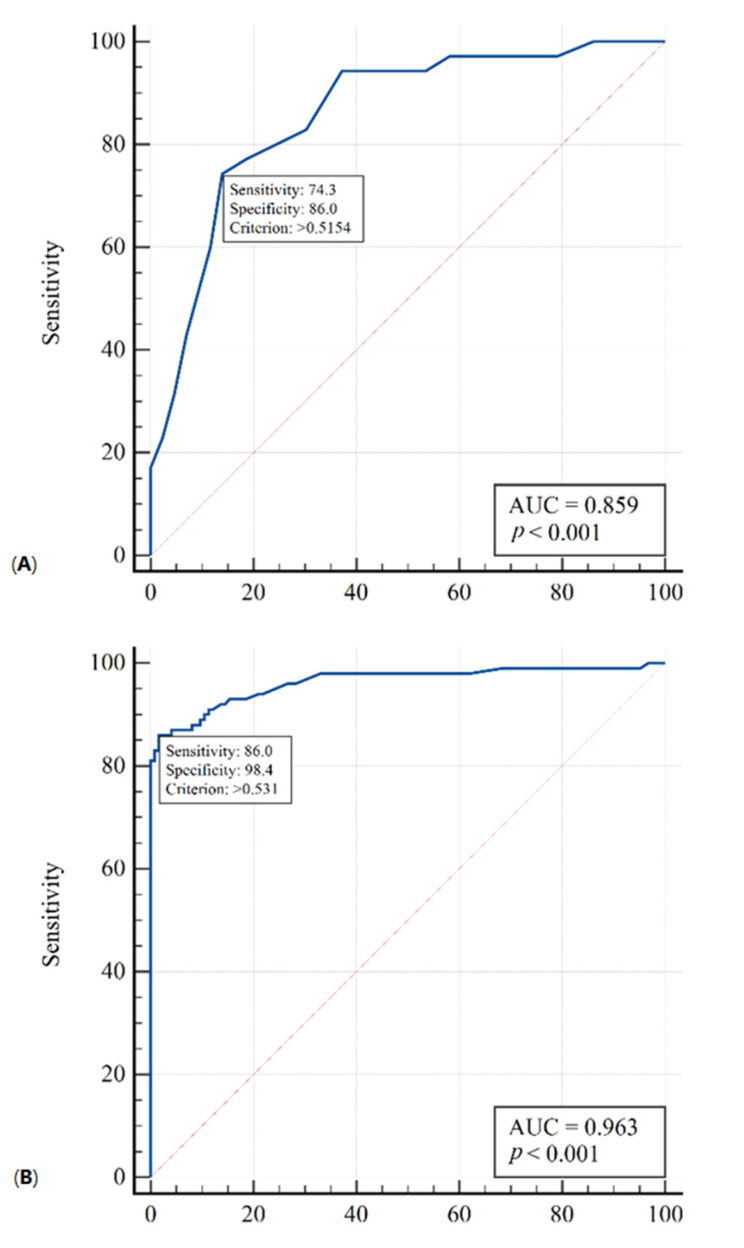
ROC curves (multivariate logistic regression models) for predicting mortality risk. (**A**): a model using procalcitonin for the Delta group. (**B**): a five-factor model using IL-6, fibrinogen, leukocytes, thrombocytes, and procalcitonin for the non-Delta group.

The models based on the selected features are adequate. As demonstrated in Figure 2, the AUCs are 0.86 (95% CI 0.76–0.93) and 0.96 (95% CI 0.93 –0.98) for the Delta and non-Delta groups, respectively. This indicates a very strong relationship between the risk of death and the selected variables in both analyses.

## 4. Discussion

To the best of our knowledge, this is the first study on the efficacy of treatment with tocilizumab in severe COVID-19 focusing on the virus variants. In our retrospective study, we did not find any statistically significant differences in mortality between patients with Delta or non-Delta variants when treated with TOC.

Assessing emerging SARS-CoV-2 mutations for the clinical severity of COVID-19 provides important information for characterising disease burden and risk to the population. The genetic diversity of the virus may affect transmissibility, virulence, and mortality, as well as the efficacy of available therapies [10]. Among the many variants of the SARS-CoV-2 virus to date, five have been recognised by the WHO as variants of concern (VOCs) of public health importance: Alpha (B.1.1.7), Beta (B.1.351), Gamma (P.1), Delta (B.1.617.2), and Omicron (B.1.1.529) [11]. The Delta variant, first detected in India in October 2020, has spread rapidly worldwide and was responsible for the deadly second wave [12]. In Ukraine, the first cases of Delta variant infection were reported in late June 2021 [13]. It was reported that the neutralising activity of vaccine- and plasma-induced neutralising antibodies of convalescents against the Delta variant was significantly reduced, which affects the risk of reinfection [14,15]. Studies on the Delta variant revealed the accumulation of nine amino acid mutations (T19R, G142D, FR156⁃157del, R158G, L452R, T478K, D614G, P681R, D950N) in the spike protein [16].

Data on the course of Delta variant infection is inconclusive. It was found that the incubation period and generation time were shorter, and the basic reproductive number was higher than that of the wild-type strain. In addition, the viral loads of the Delta variant were significantly higher. Thus, the virus showed higher transmissibility [17]. In a Scottish study, the risk of hospital admission was approximately double in the Delta compared to the Alpha variant, especially in people with multimorbidity [18]. In the study by Fisman et al. [19], the risk of hospitalisation, ICU admission, and death relative to non-VOC infections was 108%, 235%, and 133%, respectively. Lin et al. [20] showed a higher overall risk of hospitalisation rather in the Beta than the Delta variant if compared to the wild-type virus (2.16 versus 2.08, respectively). In turn, severity and mortality rates were higher in the Delta group (3.35 and 2.33 versus 2.23 and 1.50, respectively) [20]. In contrast, studies by Gunadi et al., and Tani-Sassa et al., found no significant differences in hospitalisation and mortality rates between patients with Delta and non-Delta variants [21,22].

This was in line with our results. There were no differences in mortality (44.9% versus 44.6%, *p* > 0.999), despite a significant difference in the severity of respiratory failure (PaO_2_/FiO_2_ ratio 111 versus 139 mmHg, *p* < 0.001), as well as lower lymphocyte and erythrocyte counts and a more advanced age in Delta patients (see Table 1). In a multifactor logistic regression model, three variables were found to influence mortality: erythrocyte and leukocyte counts, and procalcitonin (Table 2). There was an increased risk of death with decreasing leukocyte and erythrocyte counts and with increasing procalcitonin concentration. The analysis showed a very strong relationship between the risk of death and the selected variables with AUC = 0.93, sensitivity = 81.5%, and specificity = 94% (Figure 1).

The primary issue investigated in our paper was the impact of treatment with TOC on mortality in ICU patients with Delta or non-Delta variants. In the literature, we did not find any data on the efficacy of TOC when used for the different SARS-CoV-2 variants. In our study, no statistically significant differences in mortality were found in the Delta and non-Delta groups depending on TOC treatment (Table 4). The stepwise method found that only procalcitonin significantly influenced mortality in the Delta group, while five variables—procalcitonin along with IL-6, fibrinogen, leukocytes, and platelets—were significant for the non-Delta group (Table 5 and Table 6, Figure 2).

Initial clinical studies on the efficacy of TOC in the treatment of COVID-19 have shown inconclusive results. In a cohort study conducted at the University of Michigan, mortality among patients on artificial ventilation almost halved if treated with TOC, despite an increase in infectious complications [23]. A study from three Italian centers compared 179 patients who received TOC and 365 patients on conventional therapy. The use of TOC was associated with a lower risk of artificial ventilation or death [24]. Similarly, in a study by Capra et al., treatment with TOC was associated with improved survival and a favorable clinical course compared with standard treatment [25]. In turn, in a study by Flisiak et al., a significant effect of TOC on death was demonstrated in patients with baseline concentration of IL-6 > 100 pg/mL (OR 0.27, 95% CI 0.10–0.78, *p* = 0.02), particularly if oxygen saturation was lower than 90% [26].

Some other studies presented opposite conclusions. Campochiaro et al., compared clinical outcomes in 32 patients who received TOC and in 33 controls. Patients who received TOC had a lower mortality rate, but the differences were not statistically significant [27]. Canziani et al., demonstrated that the use of TOC was associated with a decreased probability of artificial ventilation (OR 0.36, 95% CI 0.16–0.83; *p* = 0.017), but with a higher risk of thrombosis, bleeding, or infection [28]. In addition, TOC did not affect 30-day mortality. In the COVACTA trial, the use of TOC in patients with severe COVID-19 pneumonia did not result in improved clinical status or lower mortality than the placebo at 28 days [29]. Similarly, Stone et al., conducted a randomized, double-blind, placebo-controlled trial involving 243 patients and the authors found no beneficial effect of TOC in preventing intubation or death in moderately ill hospitalized patients with COVID-19 [30].

In contrast, the results of meta-analyses published so far demonstrated the beneficial effects of TOC in the treatment of patients with severe COVID-19. Wei et al. [31] assessed 25 publications with RevMan 5.3. Significantly better clinical outcomes were found in the TOC group when compared to the standard care group with OR (odds ratio) = 0.70, 95% confidential interval (CI) 0.54–0.90, *p* = 0.007. Treatment with TOC showed a stronger correlation with a good prognosis among COVID-19 patients that needed mechanical ventilation (OR = 0.59, 95% CI 0.37–0.93, *p* = 0.02). In stratified analyses, overall mortality reduction correlated with TOC treatment in patients less than 65 years old (OR  =  0.68, 95% CI 0.60–0.77, *p*  <  0.00001), as well as in ICU patients (OR = 0.62, 95% CI 0.55–0.70, *p*  < 0.00001). In general, TOC treatment predicted better overall survival in COVID-19 patients (HR  =  0.45, 95% CI 0.24–0.84, *p* =  0.01), especially in severe cases (HR = 0.58, 95% CI 0.49–0.68, *p* < 0.00001) [31]. Maraolo et al., considered TOC to be one of the few agents that may improve the prognosis of severe COVID-19 [32]. In addition, the results of the meta-analysis by Vela et al., covering 10 RCTs and 6837 patients demonstrated a beneficial effect of TOC in patients with severe COVID-19, hyperinflammatory syndrome, and severe hypoxia. The pooled risk ratio (RR) for all-cause mortality in patients with TOC was RR = 0.88 (95% CI 0.81–0.95, *p* = 0.0009), RR for mechanical ventilation at 28–30 days was 0.79 (95% CI 0.71–0.88), and RR for serious adverse events (SAE) was 0.91, (95% CI 0.76–1.09). The reduction in mortality was most pronounced in patients who received systemic corticosteroids [33]. Data from a meta-analysis by Kyriakopoulos et al., supports recommendations for using TOC in combination with corticosteroids in patients with severe COVID-19 [34]. Tocilizumab is effective when hyperinflammatory rather than infectious mechanisms dominate the pathogenesis of the disease. The RECOVERY trial has shown that treatment with TOC in patients with severe COVID-19, hypoxemia, and hyperinflammatory syndrome reduced mortality, increased the likelihood of successful discharge from hospital, and reduced the need for invasive mechanical ventilation. These benefits are in addition to the ones provided by systemic corticosteroids [35].

A meta-analysis by Rubio-Rivas et al., comprising 64 studies covering 7668 patients with COVID-19 treated with TOC, indicated hospital-wide pooled mortality of OR 0.73 (95% CI = 0.56–0.93) but with a significant difference in efficacy depending on the severity of the disease. For patients admitted to conventional wards vs. ICU, OR were 1.25 (95% CI 0.74–2.18) and 0.66 (95% CI 0.59–0.76), respectively [36]. Again, this advantage was better seen in patients receiving concomitant corticosteroids. Another assessment covering 13 studies and 2120 patients revealed that TOC treatment resulted in lower mortality (OR = 0.42, 95% CI 0.26) as compared to standard therapy (OR = 0.69, *p* = 0.0005, I2 = 55%) [37]. Resaei et al., presented the results of 45 comparative studies involving 13,189 patients and 28 single-arm studies involving 1770 patients. Mortality (RR of 0.76 (95% CI 0.65–0.89), *p* < 0.01) and intubation risks (RR of 0.48 (95% CI 0.24–0.97), *p* = 0.04) were lower in TOC patients compared to controls, although there were no significant differences in secondary infections, length of hospital stay, hospital discharge before day 14, and ICU admission between groups [38]. A very interesting meta-analysis focused on optimal TOC use in severe and critically ill COVID-19 patients was published by Nugroho et al. [39] The authors demonstrated that TOC reduced all-cause mortality when CRP levels were ≥100 mg/L, and PaO_2_/FiO_2_ ratios were 200–300 mmHg or <200 mmHg. It is worth noting that subgroup analysis showed that TOC administration in patients with CRP levels <100 mg/L reduced neither mortality nor length of hospitalisation. Additionally, a very recently published meta-analysis involving 53 papers with 21,656 patients demonstrated a beneficial effect of TOC on mortality and intubation, but also no increase in the risk of secondary infections [40].

We cannot unequivocally justify why TOC treatment did not affect mortality in either the Delta or non-Delta groups. This may support the opinion that the use of TOC does not affect the course or mortality of COVID-19, and a possible beneficial effect can only be noted in more severe cases of the disease [31,36,39]. In our study, patients treated vs. not treated with TOC differed slightly, although those differences were similar in the Delta vs. non-Delta groups. Those treated with TOC in the Delta group had higher D-dimer and ferritin levels and lower erythrocyte and lymphocyte counts while patients with the non-Delta virus variant had higher D-dimer, fibrinogen, and IL-6 levels and lower erythrocyte and lymphocyte counts (Table 3). This may indicate a more severe course of COVID-19 in the TOC-treated groups, but it seems doubtful that these differences are clinically relevant.

Another possible explanation may be COVID-19 heterogeneity. In our previous study, TOC contributed to reduced mortality in patients with macrophage activation syndrome [41]. In contrast, in those with cytokine release syndrome, TOC even increased mortality. The small size of the groups studied did not allow for such a subanalysis. Thus, we believe that TOC efficacy depends on COVID-19 severity and the cytokine storm subtype rather than the virus variant.

It should be also noted that Delta and non-Delta patients were not hospitalised at the same time. Thus, it cannot be excluded that the final outcome could be influenced by the centres experience with COVID-19 treatment, availability of beds, or faster qualification for ICU treatment.

Our study has limitations. Firstly, it is a retrospective, single-centre study. Secondly, a low number of patients in the study groups significantly limits the power of this study and its conclusions. Finally, the study concerned a specific virus variant that is no longer dominant, thus there is a question of whether the results obtained can be extrapolated to other SARS-CoV-2 variants.

## 5. Conclusions

Tocilizumab did not improve survival in patients with both Delta and non-Delta variants. Despite a more severe clinical course, the Delta variant did not increase mortality in COVID-19.

## Figures and Tables

**Table 1:** Basic demographic and medical data of the groups studied. Median (Q_I_-Q_III_) for continuous variables, numerical values (%) for categorical variables. The Mann–Whitney test was used for comparison between continuous variables, and Fisher’s exact test was used for categorical variables.

	Delta (*n* = 78)	Non-Delta (*n* = 224)	*p* Value
Age, years	70 (68–72)	68 (66–71)	<0.001
Sex, female (%)	37 (47.4)	108 (48,2)	0.896
Temperature, ^°^C	37.5 (37.2–37.8)	37.5 (37.3–37.8)	0.947
CRP, mg/L	113 (109–121)	114 (101.5–123.5)	0.825
Interleukin-6, pg/mL	64 (47–71)	60 (47–72)	0.537
Procalcitonin, ng/mL	0.8 (0.5–1.1)	0.8 (0.6–1.0)	0.695
Ferritin, ng/mL	1211.5 (1011–1478)	1216.5 (1033.5–1349)	0.457
Fibrinogen, g/L	6.7 (6.6–6.9)	6.8 (3.5–6.9)	0.320
D-dimer, µg/L	1422 (1215–1977)	1341 (1233–1647)	0.081
Leukocytes, × 10^9^/L	4.2 (3.5–4.3)	4.2 (3.950–4.3)	0.934
Lymphocytes, %	22 (17–24)	24 (22–26)	<0.001
Thrombocytes, × 10^9^/L	126 (101–138)	128 (102–138)	0.609
Erythrocytes, × 10^12^/L	2.6 (2.2–3.2)	3.2 (2.6–3.7)	<0.001
PaO_2_/FiO_2_, mm Hg	111 (74–130)	139 (82–152)	<0.001
Treatment with TOC, (%)	30 (38.5)	84 (37,5)	0.893
Mortality, (%)	35 (44.9%)	100 (44.6%)	>0.999

Laboratory test reference ranges: CRP < 5.0 mg/L, interleukin-6 <4.0 pg/mL, procalcitonin < 0.02 ng/mL, ferritin 8–143 ng/mL, fibrinogen 2.0–4.0 g/L, D-dimer <5 00 µg/L, leukocytes 4.0–9.0 × 10^9^/L, lymphocytes 19–37%, thrombocytes 200–400 × 10^9^/L, erythrocytes 3.6–4.2 × 10^12^/L, PaO_2_/FiO_2_ 454–495 mm Hg.

**Table 2:** Analysis of risk of death in a three-factor logistic regression model.

Independent Variables	The Model Coefficient, b ± m	Significance Level of Difference of the Coefficient from 0, *p* Value	OR (95% CI)
Procalcitonin, ng/mL	7.788 ± 0.97	<0.001	2646 (392–18,000)
Erythrocytes, × 10^12^/L	−0.98 ± 0.31	0.002	0.38 (0.20–0.99)
Leukocytes, × 10^9^/L	−0.26 ± 0.13	0.038	0.77 (0.60–1.003)

**Table 3:** Basic demographic and medical data of the study groups with respect to treatment with tocilizumab. Median (Q_I_–Q_III_) for continuous variables, numerical values (%) for categorical variables. The Mann–Whitney test was used for comparison between continuous variables, and Fisher’s exact test was used for categorical variables.

A—Delta Group	no-TOC (*n* = 48)	TOC (*n* = 30)	*p* Value
Age, years	71 (68–72)	70 (68–71)	0.552
Sex, female (%)	22 (45.8)	15 (50)	0.817
Temperature, ^°^C	37.5 (37.2–37.75)	37.6 (37.5–37.9)	0.111
CRP, mg/L	113 (106–121)	112.5 (109–123)	0.861
Interleukin-6, pg/mL	60 (32.5–72)	64 (61–68)	0.36
Procalcitonin, ng/mL	0.8 (0.2–1.1)	0.8 (0.7–1.2)	0.077
Ferritin, ng/mL	1117.5 (998–1313.5)	1483 (1143–1789)	<0.001
Fibrinogen, g/L	6.7 (6.6–6.9)	6.7 (6.6–7.6)	0.203
D-dimer, µg/L	1245 (1121–1423)	2108.5 (1657–2513)	<0.001
Leukocytes, × 10^9^/L	4.2 (3.5–4.3)	4.2 (4–4.3)	0.36
Lymphocytes, %	24 (22–26)	17 (16–18)	<0.001
Thrombocytes, × 10^9^/L	128 (102–138)	126 (85–128)	0.261
Erythrocytes, × 10^12^/L	3.0 (2.6–3.65)	2.2 (2.2–2.3)	<0.001
PaO_2_/FiO_2_, mm Hg	111 (76.5–132.5)	98 (71–120)	0.051
**B—Non-Delta group**	**no-TOC (*n* = 140)**	**TOC (*n* = 84)**	***p* value**
Age, years	68 (66–70)	69 (66.5–71)	0.062
Sex, female (%)	68 (48.6)	40 (47.6)	>0.999
Temperature, ^°^C	37.5 (37.2–37.7)	37.5 (37.4–37.95)	0.036
CRP, mg/L	113 (103.5–122.5)	115 (99–125.5)	0.391
Interleukin-6, pg/mL	60 (34–72)	68 (49–76)	0.018
Procalcitonin, ng/mL	0.8 (0.7–1.1)	0.8 (0.5–1.0)	0.073
Ferritin, ng/mL	1211.5 (1015.5–1323.5)	1222.5 (1067–1454.5)	0.239
Fibrinogen, g/L	6.5 (3.5–6.9)	6.8 (6.650–8.6)	<0.001
D-dimer, µg/L	1305 (1211–1423)	1432 (1341–1763)	<0.001
Leukocytes, × 10^9^/L	4.1 (3.5–4.2)	4.2 (4.2–4.35)	<0.001
Lymphocytes, %	24 (23–26)	23 (18–26)	0.006
Thrombocytes, × 10^9^/L	126 (102–138)	128 (85–138)	0.96
Erythrocytes, × 10^12^/L	3.2 (2.75–3.7)	2.8 (2.6–3.3)	0.014
PaO_2_/FiO_2_, mm Hg	136.5 (81–152)	142 (88–149.5)	0.070

**Table 4:** Death n (%) in Delta and non-Delta patients treated or not with tocilizumab (TOC, no-TOC); Fisher’s exact test was used.

		TOC	No-TOC	*p* Value
**Delta group**				
Death	No	16 (53.3%)	27 (56.2%)	0.819
	Yes	14 (46.7%)	21 (43.7%)	
**Non-Delta group**				
Death	No	52 (61.9%)	72 (51.4%)	0.165
	Yes	32 (38.1%)	68 (48.6%)	

**Table 5:** Analysis of risk of death in a single-factor logistic regression model for the Delta group.

Independent Variables	The Model Coefficient, b ± m	Significance Level of Difference of the Coefficient from 0, *p* Value	OR (95% CI)
Procalcitonin, ng/mL	4.03 ± 0.91	<0.001	56.5 (9.4–341)

**Table 6:** Analysis of risk of death in a five-factor logistic regression model for the non-Delta group.

Independent Variables	The Model Coefficient, b ± m	Significance Level of Difference of the Coefficient from 0, *p* Value	OR (95% CI)
Leukocytes, × 10^9^/L	–0.541 ± 0.21	0.015	0.60 (0.40–0.91)
Fibrinogen, g/L	1.23 ± 0.27	<0.001	3.43 (2.03–5.80)
IL-6, pg/mL	−0.032 ± 0.016	0.040	0.97 (0.94–0.99)
Thrombocytes, × 10^9^/L	−0.063 ± 0.019	0.001	1.07 (1.03–1.10)
Procalcitonin, ng/mL	13.3 ± 2.1	<0.001	5 × 10^5^ (9900–3.4 × 10^6^)

## Data Availability

All relevant data is provided in the manuscript.
